# Complete mitochondrial genome of the Arctic hare, *Lepus arcticus*

**DOI:** 10.1080/23802359.2019.1677193

**Published:** 2019-10-16

**Authors:** Bo-Mi Kim, Won Young Lee, Jae-Sung Rhee

**Affiliations:** a Unit of Polar Genomics, Korea Polar Research Institute, Incheon, South Korea;; b Division of Polar Life Sciences, Korea Polar Research Institute, Incheon, South Korea;; c Department of Marine Science, College of Natural Sciences, Incheon National University, Incheon, South Korea;; d Research Institute of Basic Sciences, Incheon National University, Incheon, South Korea

**Keywords:** North Greenland, Arctic hare, *Lepus arcticus*, Lagomorph, mitogenome

## Abstract

In this study, we report on the complete mitochondrial genome of the Arctic hare, *Lepus arcticus* (Leporidae; Lagomorpha) a large lagomorph endemic to the northernmost regions of Greenland. The complete mitogenome of *L. arcticus* was 16,972 bp long and was typical of genus *Lepus* mitogenomes in genomic content and structure, as the entire mitogenome contained 13 protein-coding genes (PCGs), two ribosomal RNA (rRNA) genes, 22 transfer RNA (tRNA) genes, and one control region. The phylogenetic analysis of the Arctic hare within Leporidae confirmed the sister relationship among *Lepus* species. This mitogenome sequence will provide a useful resource for investigations of biogeography, phylogenetic distance, and evolutionary history in lagomorphs.

The order Lagomorpha comprises Leporidae (hares, rabbits) and Ochotonidae (pikas), and the genus *Lepus* unifies cosmopolitan hares. The Arctic hare *Lepus arcticus* is distributed from Greenland and Canadian Arctic islands to parts of the Canadian mainland east of the Mackenzie River and north of the tree line (Best and Henry 1994; Dalerum et al. 2017). Major differences of *L. arcticus* are larger in body weight (3.5–6.0 kg at adult stage) and heavily padded feet with strong front and hind claws than other hare species (Howell 1936; Best and Henry 1994). Previously, population density was reported from Newfoundland as ≈1 hare per km^2^ (Mercer et al. 1981; Hearn et al. 1987) and irregular temporal fluctuations in population size have been reported (Banfield 1951; Parker 1977; Mech 2005; Dalerum et al. 2017). Three species of Arctic or northern hare has been recognised as the Arctic hare (*L. arcticus*), Alaskan hare (*L. othus*; western and northwestern regions of Alaska), and the Mountain hare (*L. timidus*; the Palaearctic region from Great Britain and Fennoscandia to eastern Siberia), but overall taxonomic status on the genus *Lepus* is still controversial due to morphological characters, insufficient geographical survey, overlap of distributional ranges, uncertain source for each species, introgression, complex evolutionary history, and lack of molecular information (Robinson and Matthee 2005; Waltari and Cook 2005; Alves et al. 2008; Melo-Ferreira et al. 2012; Ge et al. 2013). Therefore, accumulation of mitogenome information on the genus *Lepus* will be helpful to understand molecular phylogeny and genetic diversity of leporids.

In this study, we assembled the entire mitogenome of *L. arcticus* (Accession no. MK948870) by employing Illumina HiSeq platform (Illumina, San Diego, CA). Tissue sample was isolated from the foreleg of dead individual of *L. arcticus* at Sirius Passet (82°47′4.3″N, 42°27′11.6″W; Lee 2018) on 11 July 2017. The voucher specimen was deposited in the Korea Polar Research Institute (KOPRI; Species ID: AH; Specimen ID: 170711_AH01). Extraction of the total genomic DNA was performed using DNeasy Blood and Tissue kit (Qiagen, Hilden, Germany), followed by sequencing library generation with TruSeq RNA Sample Preparation Kit according to the manufacturer's instructions (Illumina) and paired-end sequencing on Illumina HiSeq platform (Illumina) at Phyzen (Seoul, South Korea). Adapter sequences, low quality reads (sequences with >50% bases with quality value ≤5), reads with >10% of unknown bases, and ambiguous bases were totally removed to obtain high quality reads (Phred score of >20). CLC Assembly Cell package (version 4.2.1) with the CLC de novo assemble algorithm was used for assembly. Additional PCR procedure and Sanger sequencing was conducted to confirm the nucleotide sequence of control region. Entire *L. arcticus* mitogenome were annotated by using the MITOS web-based software (Bernt et al. 2013) and detailed annotation were conducted with NCBI-BLAST (http://blast.ncbi.nlm.nih.gov).

The complete mitochondrial genome of *L. arcticus* was 16,972 bp in length and contained the typical set of 13 PCGs, 22 tRNAs, two rRNAs, and one control region, located in the arrangement typical of leporid mitogenomes. For 10 out of the 13 PCGs of *L. arcticus*, the traditional mitochondrial open reading frame stop codons was used for termination, while *COX3*, *ND3*, and *ND4* genes had incomplete stop codon. A phylogenetic analysis was constructed using the nucleotide sequence of cytb gene of *L. arcticus* with including of 15 published mitogenomes from Leporidae or Ochotonidae (Figure 1). We used JModelTest ver. 2.1.10 (Darriba et al. 2012) to select the best substitution model and a substitution model (HKY + G + I) was employed to construct a maximum-likelihood (ML) method in the PhyML 2.4.5 (Guindon and Gascuel 2003) with 1000 bootstrap replicates. Phylogenetic relationship showed that *L. arcticus* grouped together with other representatives of Leporidae. The sequence of *L. othus* was excluded from the analysis due to incomplete PCGs registered in GenBank, although a highly supported clade has been recovered for *L. arcticus*, *L. othus*, and *L. timidus* with partial gene set (Waltari and Cook 2005; Alves et al. 2008; Melo-Ferreira et al. 2012; Ge et al. 2013). In conclusion, the complete *L. arcticus* mitogenome will provide useful information to elucidate phylogenetic relationship, geographical distribution, and evolution of the family Leporidae.

**Figure 1.**
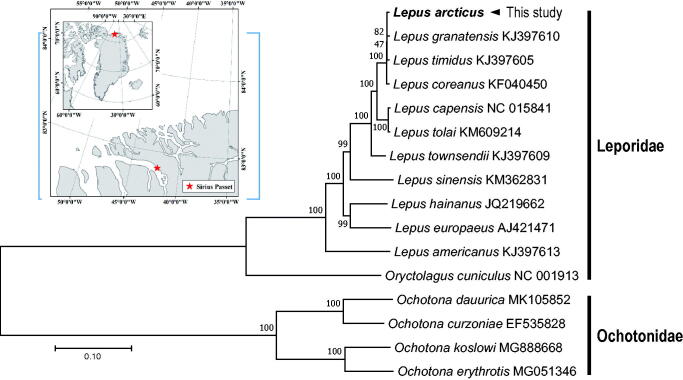
Maximum-likelihood (ML) phylogeny of 12 species of the Leporidae family including 11 leporids based on the nucleotide sequences of cytb gene. Four species from the family Ochotonidae were used as outgroup. Numbers on the branches indicate ML bootstrap percentages (1000 replicates). DDBJ/EMBL/Genbank accession numbers for published sequences are incorporated. Small box represents the sampling site in Greenland.
